# Supplementary material for: Sustained-input switches for transcription factors and microRNAs are central building blocks of eukaryotic gene circuits
Source: Genome Biol. 2013 Aug 23;14(8):R85. doi: 10.1186/gb-2013-14-8-r85 (PMC4054853; doi:10.1186/gb-2013-14-8-r85)
Supplement: Additional file 5 — HTML Browsable Motif Output. Zipped folder containing all WaRSwap and FANMOD motif output, viewable in a web browser. [file gb-2013-14-8-r85-S5.ZIP › HTML_browsable_motif_output/WaRSwap_all_tair9/sigs_WSWRSLDraw-2000_all.pvals.heatmaps.html/motif_id_14_011110001_tftype_all_upstream_-3000_0.html]

```
BG_MODEL = Rnew2
MOTIF_ID = 14_011110001
TF_TYPE = all
UPSTREAM = -3000_0


PVals
FN_0.2	FN_0.4	FN_0.6	FN_0.8
dg_60.genes	0.358	0.007	0.015	0.761
dg_70.genes	0.273	0.002	0.013	0.732
dg_80.genes	0.711	0.134	0.118	0.604

ZScores
FN_0.2	FN_0.4	FN_0.6	FN_0.8
dg_60.genes	0.352	2.81	2.828	-0.948
dg_70.genes	0.635	3.176	3.041	-0.877
dg_80.genes	-0.644	1.164	1.065	-0.773

StDevs
FN_0.2	FN_0.4	FN_0.6	FN_0.8
dg_60.genes	729.893	394.137	131.684	21.07
dg_70.genes	707.242	371.353	125.981	21.149
dg_80.genes	571.033	306.98	113.595	16.308
```
